# Animal Models of Hunner‐Type Interstitial Cystitis

**DOI:** 10.1111/iju.70487

**Published:** 2026-05-01

**Authors:** Yoshiyuki Akiyama, Yi Luo

**Affiliations:** ^1^ Department of Urology Shinshu University School of Medicine Matsumoto Nagano Japan; ^2^ Department of Urology University of Iowa Iowa City Iowa USA

**Keywords:** animal model, bladder pain syndrome, Hunner‐type IC, IC/BPS, interstitial cystitis

## Abstract

**Background:**

Animal models are crucial for mechanistic studies and therapeutic development of human diseases. At present, the etiology of interstitial cystitis/bladder pain syndrome (IC/BPS), a chronic disease of the urinary bladder, remains undefined. Therefore, numerous theories of pathogenesis have been proposed, and various animal models have been developed based on these theories. This enigmatic human disease can be categorized into two subtypes: Hunner‐type IC (HIC) and bladder pain syndrome (BPS). These two subtypes of IC/BPS have different pathological mechanisms, but their clinical symptoms overlap. Recent evidence indicates that HIC is an immune‐mediated inflammatory disease of the urinary bladder, while BPS is a minimally inflamed bladder condition comprising various clinical phenotypes. Furthermore, increasing evidence suggests that autoimmunity may play a significant role in IC/BPS, particularly in HIC. Today, the rodent models of experimental autoimmune cystitis (EAC) are being used in HIC research.

**Objective:**

This article provides an overview of immune‐mediated inflammation and autoimmunity in IC/BPS, as well as EAC models that can be used for HIC research, with a focus on the URO‐OVA model, a novel transgenic EAC model that effectively mimics HIC.

**Conclusion:**

The URO‐OVA model develops chronic bladder inflammation, pelvic/bladder pain, and voiding dysfunction seen in human HIC patients. It responds to treatment with dimethyl sulfoxide (DMSO) and specific inhibitors, such as Toll‐like receptor (TLR)4, mitogen‐activated protein (MAP) kinase, and interferon (IFN)‐γ inhibitors. The URO‐OVA model is stable and reproducible, providing a unique EAC model for HIC research that incorporates immune/autoimmune components in its pathophysiology.

## 1 Introduction

**FIGURE 1:**
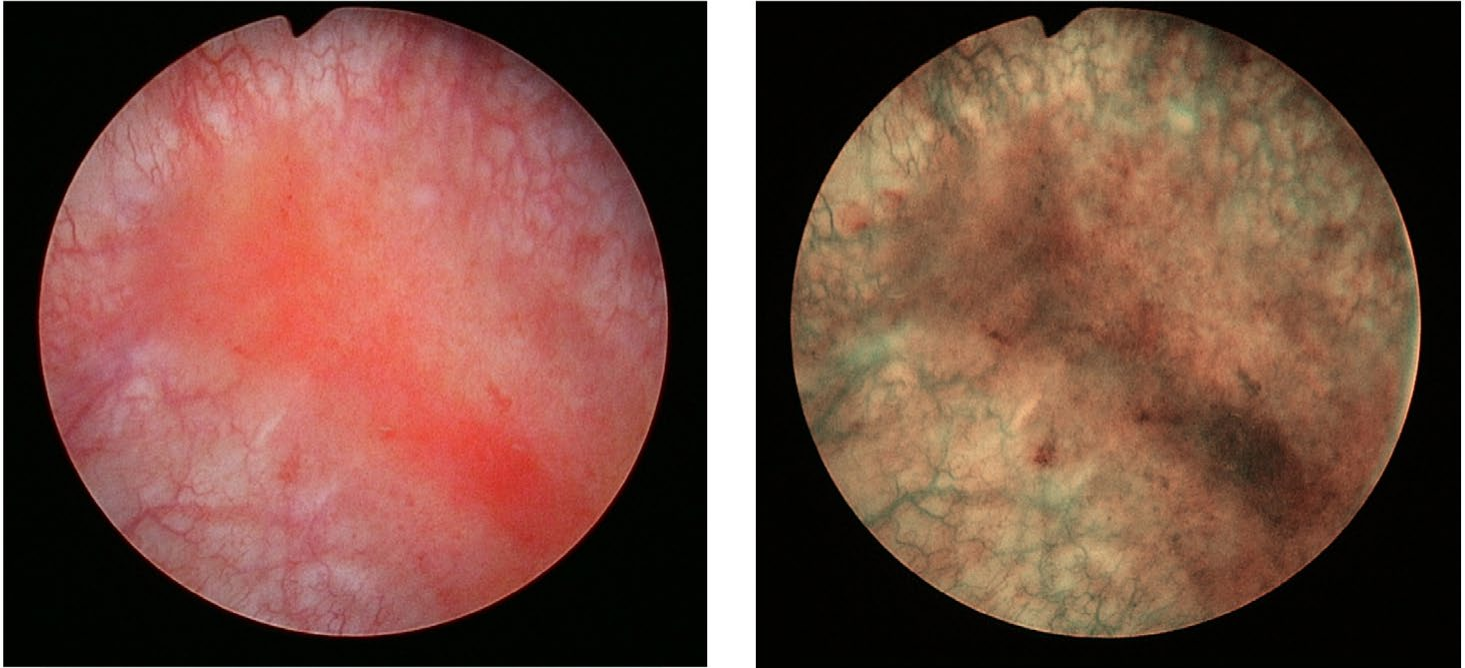
A Hunner lesion. (Left) A Hunner lesion is a reddish mucosal lesion accompanied by abnormal capillary structures. (Right) Narrow‐band imaging cystoscopy of the Hunner lesion emphasizes the abnormal capillary structures converging toward the lesion.

Interstitial cystitis/bladder pain syndrome (IC/BPS) is a chronic and debilitating urologic disorder characterized by the hallmark symptom of persistent pelvic/bladder pain in the absence of other identified etiologies for the symptom [1, 2]. Patients with IC/BPS also suffer from lower urinary tract symptoms (LUTS) such as excessive urinary frequency and/or urgency [1, 2]. This urologic condition is significant and severely affects patients' quality of life [3]. Its financial burden on the US healthcare system is substantial [4]. The prevalence of IC/BPS is 2.7%–6.53% in U.S. women (3–8 million at age 18 or older) [5] and 2.9%–4.2% in U.S. men [6], respectively. The prevalence of IC/BPS in Asian countries ranges from 0.022% to 0.045% in female patients [7]. The male‐to‐female ratio is 1:5.8 in Japan [8]. The prevalence of IC/BPS increases with age [7, 9]. This enigmatic syndrome can be categorized into two subtypes: Hunner‐type IC (HIC), which is characterized by the cystoscopic presence of a Hunner lesion, a reddish and hemorrhagic mucosal lesion accompanied by abnormal capillary structures (Figure 1), and bladder pain syndrome (BPS) [10, 11, 12]. The two IC/BPS subtypes present significantly overlapping clinical manifestations and cannot be distinguished on a symptomatology basis [10, 11, 12]. Accumulating evidence indicates that HIC is an immune‐mediated inflammatory disease of the urinary bladder histologically characterized by dense infiltrating lymphocytes, increased plasma cell and mast cell counts, and urothelial denudation, while BPS is a minimally inflamed bladder condition comprising various clinical phenotypes. The latter has less mononuclear cell infiltration in the bladder and intact urothelium. HIC presents in 10%–20% of all IC/BPS patients in North American population [13, 14], but the worldwide prevalence of HIC has varied widely from 5% to 57% [13, 15, 16]. At present, there is no cure for IC/BPS. All available treatments are largely aimed at easing symptoms with variable efficacy due to the various clinical phenotypes of IC/BPS and the heterogenous patient populations. Therefore, animal models are crucial for studying the mechanisms of IC/BPS and for developing new therapies for IC/BPS.

Since the etiology of IC/BPS remains undefined, numerous theories of pathogenesis have been proposed including increased urothelial permeability, chronic inflammation, immune dysregulation, autoimmunity, neurogenic hyperactivity, and psychological stress. Accordingly, a wide variety of animal models have been developed to recapitulate the complex pathophysiology of IC/BPS (Table 1) [10, 61, 62]. Collectively, these IC/BPS animal models can be classified into “Increased urothelial permeability models” [17, 18, 19], “Bladder inflammation models” [20, 21, 22, 23, 24, 25, 26, 27, 28, 29, 30], “Experimental autoimmune cystitis (EAC) models” [31, 32, 33, 34, 35, 36, 37, 38, 39, 40, 41, 42, 43, 44, 45, 46, 47, 48, 49, 50, 51, 52, 53], “Psychological and physical stress models” [54, 55, 56], “Pelvic cross‐organ sensitization models” [57, 58], and “Naturally occurring model” such as IC in feline [59, 60]. These animal models can be generated through (i) Bladder instillation (i.b.) of protamine sulphate (PS), hydrochloric acid (HCl), hydrogen peroxide (H_2_O_2_), lipopolysaccharide (LPS), chondroitinase ABC/haparanase III, zymosan and hyaluronidase; (ii) Intraperitoneal (i.p.) injection of cyclophosphamide (CYP); (iii) Subcutaneous (s.c.) immunization with bladder homogenate, uroplakin protein (UPK) or UPK peptide; (iv) Transgenic technology that enables the urothelial expression of monocyte chemoattractant protein‐1 (MCP‐1, a CC chemokine), ovalbumin (OVA, a model antigen) or tumor necrosis factor (TNF, a proinflammatory cytokine); (v) Water avoidance and neonatal maternal separation; (vi) Intracolonic application of trinitrobenzene sulfonic acid (TNBS) and peritoneal implantation of uterine tissue; (vii) Naturally occurring seen in feline IC. Based on their pathophysiological characteristics, these animal models can be divided into HIC‐like models or BPS‐like models [61]. Each of these animal models resembles certain key features of IC/BPS such as pelvic/bladder pain, voiding dysfunction, histological inflammation, and/or urothelial damage. In addition to these symptom‐ and pathologically related components, some animal models can also capture potential specific pathogenic triggers such as infection, autoimmunity and chronic stress. Despite the many advantages of these animal models, each model has its specific limitations and may not fully capture the complexity of IC/BPS. For example, most models lack spontaneous pain behavior, a few models demonstrate bowel‐bladder interactions, and chemically induced models fail to capture the chronic characteristics of IC/BPS. Currently, rodent EAC models are used for HIC research because these models develop chronic bladder inflammation and symptoms similar to those of human HIC [31, 52, 53, 61, 62]. In this article, we discuss immune‐mediated inflammation and autoimmunity in IC/BPS and review EAC models that can be used for HIC research, with a focus on our developed URO‐OVA model, a novel transgenic EAC model that effectively mimics HIC.

**TABLE 1:** Animal models of IC/BPS.

Model types	Induction methods	HIC‐ and BPS‐related findings	Model limitations
Increased urothelial permeability	i.b. PS [17, 18] i.b. chondroitinase ABC/heparanase III [19]	Limited inflammation, edema, urothelial damage, bladder hyperpermeability, bladder afferent hypersensitivity Neutrophilic infiltration, mast cell accumulation, urothelial damage, frequent voiding, pelvic nociception	Nociception was not fully evaluated. Urinary frequency was not evaluated Bladder nociception was not evaluated. Unconscious cystometry was used
Bladder inflammation	i.b. HCl [20] i.b. H_2_O_2_ [21] i.b. hyaluronidase [22] i.b. Zymosan [23] i.b. LPS [24, 25] i.p. CYP [26, 27] Transgenic URO‐MCP‐1 [28, 29] Transgenic UPII‐TNF [30]	Mast cell and eosinophilic accumulation, edema, bladder thickening, frequent voiding Neutrophilic infiltration, edema, overexpression of TNF‐α, IL‐1β and IL‐6, urothelial thinning, bladder hyperpermeability, frequent voiding Mast cell accumulation, fibrosis, overexpression of IL‐6 and ICAM‐1, urothelial thinning, frequent voiding, perineal nociception Increased plasma extravasation, bladder nociception Leukocyte infiltration, edema, hemorrhage, fibrosis, urothelial damage, frequent voiding, pelvic nociception Edema, hemorrhage, urothelial damage, bladder wall thickness, overexpression of IL‐1β, IL‐6 and MCP‐1, frequent voiding, pelvic nociception Macrophage infiltration, edema, overexpression of IL‐1β, IL‐6, SP and NGF, bladder hyperpermeability, frequent voiding, pelvic nociception Overexpression of TNF and CGRP, urothelial damage, frequent voiding, pelvic nociception, bladder afferent hypersensitivity	Nociception was not evaluated. Unconscious cystometry was used Nociception was not evaluated Pelvic/bladder nociception was not evaluated. Unconscious cystometry was used Pelvic nociception and urinary frequency were not evaluated Bladder nociception was not evaluated. Unconscious cystometry was used Bladder nociception was not evaluated Bladder nociception was not evaluated Bladder nociception was not fully evaluated
Experimental autoimmune cystitis	s.c. bladder homogenate [31, 32, 33, 34, 35, 36, 37, 38] s.c. uroplakin or peptide [39, 40, 41, 42] s.c. TRPM8 T2 peptide [43] FcγRIIb and PD‐1 double knockout mice [44] Transgenic URO‐OVA [45, 46, 47, 48, 49, 50, 51, 52, 53]	Lymphocytic and neutrophilic infiltrations, mast cell accumulation, edema, fibrosis, glomerulation, overexpression of IFN‐γ, IL‐1β, IL‐6 and TNF‐α, urothelial damage and denudation, bladder hyperpermeability, frequent voiding, pelvic nociception T cell infiltration, mast cell accumulation, overexpression of IFN‐γ, TNF‐α, IL‐1β, IL‐2 and IL‐17, frequent voiding, pelvic nociception T cell infiltration, mast cell accumulation, edema, overexpression of TNF‐α, frequent voiding, pelvic nociception Infiltration of T cells, B cells and dendritic cells, mast cell accumulation, urothelial damage, overexpression of TNF‐α and IL‐1β, reduced urine volume per void Infiltration of CD8^+^ T cells, CD4^+^ T cells and CD19^+^ B cells; mast cell accumulation, edema, hyperemia, overexpression of IFN‐γ, TNF‐α, IL‐6, NGF, SP and MCP‐1, frequent voiding, pelvic/bladder nociception	Bladder nociception was not evaluated. Autoantigens are unknown Bladder nociception was not evaluated Bladder nociception was not evaluated Nociception and urinary frequency were not evaluated OVA is not a natural urothelial autoantigen
Psychological/physical stress	Water avoidance [54, 55, 56] Neonatal maternal separation [56]	Mast cell accumulation and degranulation, urothelial damage, frequent voiding, nociceptive behavior, bladder nociception Mast cell accumulation and degranulation, overexpression of IL‐6, IL‐10 and NGF, frequent voiding, bladder nociception	Pelvic nociception was not evaluated. Unconscious cystometry was used Pelvic nociception was not evaluated
Pelvic cross‐organ sensitization	i.b. PS or i.c. TNBS [17, 57] Peritoneal implantation of uterine tissue [58]	Bladder/colon hyperpermeability. i.c. TNBS also induced colonic inflammation and damage, nociceptive behavior, overexpression of TRPV1, increased activity of MPO Overexpression of TRPA1, frequent voiding	Pelvic/bladder nociception and urinary frequency were not evaluated Nociception and inflammation were not evaluated
Feline IC	Naturally occurring [59, 60]	Neutrophilic infiltration, mast cell accumulation, edema, vasodilation, hemorrhage, urothelial damage and denudation, Hunner's ulcers, bladder hyperpermeability, frequent voiding, nociceptive behavior	Nociception has not been fully evaluated. Equal sex distribution (This does not reflect the preponderance of women in IC/BPS)

Abbreviations: CGRP, calcitonin gene‐related peptide; CYP, cyclophosphamide; H_2_O_2_, hydrogen peroxide; HCl, hydrochloric acid; i.b., bladder instillation; IBS, irritable bowel syndrome; i.c., intracolonic infusion; ICAM‐1, intercellular adhesion molecule‐1; IFN, interferon; IL, interleukin; i.p., intraperitoneal injection; LPS, lipopolysaccharide; MCP, monocyte chemotactic protein; MPO, myeloperoxidase (a proinflammatory factor); NGF, nerve growth factor; OVA, ovalbumin; PS, protamine sulphate; s.c., subcutaneous injection; SP, substance P precursor; Th1, T helper type 1; TNBS, trinitrobenzene sulfonic acid; TNF, nerve growth factor; TRPA1, Transient receptor potential ankyrin 1; TRPV1, transient receptor potential vanilloid 1 (a pain‐signaling receptor); UPII, uroplakin II gene promoter; URO, uroplakin II gene promoter.

## 2 Immunoreactivity and Inflammation in IC/BPS

The urinary bladder is a well‐known immunoreactive organ, prone to both infectious and non‐infectious inflammations. Urinary tract bacteria (e.g., *E coli*) can cause infectious bladder inflammation such as acute urinary tract infections (UTIs). Non‐infectious bladder inflammation can be a complication of systemic diseases outside of the bladder or can be caused by an inflammatory disease of the bladder itself, such as HIC [63]. Considerable data have been published on bladder, urine and serum specimens from patients with IC/BPS, indicating a role of cell‐mediated immune mechanisms in IC/BPS, particularly in HIC (Table 2) [10, 11, 64, 65, 66, 67, 68, 69].

**TABLE 2:** Immunoreactivity and inflammation in IC/BPS.

	HIC	BPS	Unspecified
Bladder histology	Lymphoid follicles, dense T and B (plasma) cells, increased mast cells, urothelial denudation	Minimal inflammation, few plasma cells, rare mast cells, intact urothelium	
Potential inflammatory factors			
Bladder	IL‐17A, BAFF, HIF1α, NO, TLR7, IFN‐γ, CXCR3, VEGF	NGF	UPIII, E‐Cadherin, ZO‐1, CXCL1, CXCL8, CXCL9, CXCL11, IL‐6, IL‐7, IL‐8, MIP‐1β, Eot, RANTES
Urine	CXCL10, VEGF	NGF PD‐ECGF	APF, CCL2, EGF, HB‐EGF, MMP‐9, TNF‐α, UPIII, CXCL1, CXCL8, IL‐2, IL‐4, IL‐6, IL‐7, IL‐8, IL‐9, PGE_2_, MCP‐1, MIP‐1β, Eot, RANTES, MIF
Serum	VEGF	NGF	TNF‐α, UPIII, IL‐1β, IL‐4, IL‐6, IL‐8, IgE, MIP‐1β

Abbreviations: APF, antiproliferative factor; BAFF, B‐cell activating factor; BPS, bladder pain syndrome; CCL, C‐C motif ligand; CXCL, CXC chemokine ligand; CXCR, CXC chemokine receptor; E2; MCP, monocyte chemotactic protein; EGF, epidermal growth factor; Eot, eotaxin; HB‐EGF, heparin‐binding EGF‐like growth factor; HIC, Hunner‐type interstitial cystitis; HIF, hypoxia‐inducible factor; IFN, interferon; Ig, immunoglobulin; IL, interleukin; MIF, macrophage migration inhibitory factor; MIP, macrophage inflammatory protein; MMP, matrix metalloproteinase; NGF, nerve growth factor; NO, nitric oxide; PD‐ECGF, platelet‐derived endothelial cell growth factor; PGE_2_, prostaglandin; RANTES, regulated upon activation normal T cell expressed and secreted; TLR, toll‐like receptor; TNF, tumor necrosis factor; UP, uroplakin; UPIII, uroplakin III; VEGF, vascular endothelial growth factor.

The bladder histology of HIC shows robust subepithelial chronic inflammation characterized by dense T and B lymphocyte infiltrations frequently accompanied by lymphoid follicles/aggregates, increased mast cell counts, and urothelial denudation within the Hunner lesions and in background mucosa outside the lesions. The observed dense T and B lymphocytes including plasma cells in the HIC bladder suggest a possible role of adaptive immunity in HIC. In contrast to HIC, the bladder of BPS exhibits minimal inflammation, few infiltrating mononuclear cells, and intact urothelium [67]. In addition to bladder histopathology, various inflammatory factors have been found in the bladder, urine and serum of patients with IC/BPS [67, 68, 69]. It is noteworthy that some inflammatory factors appear to be specific to or predominantly associated with HIC, for example, interleukin (IL)‐17A, B cell activating factor (BAFF), hypoxia‐inducible factor 1‐alpha (HIF1α) and interferon (IFN)‐γ, while others appear to be specific to or predominantly associated with BPS, for example, nerve growth factor (NGF) and platelet‐derived endothelial cell growth factor (PD‐ECGF) [53, 70, 71, 72, 73, 74, 75, 76, 77, 78, 79, 80, 81]. In addition, numerous inflammatory factors have been found in patients with unspecified IC/BPS. These aberrantly expressed inflammatory factors may have the potential to serve as diagnostic biomarkers and/or therapeutic targets for IC/BPS. Further research to identify key inflammatory factors in HIC versus BPS will greatly improve the clinical management of IC/BPS.

## 3 Autoimmune Inflammation in IC/BPS

Autoimmunity has been suggested to play a significant role in the pathophysiology of IC/BPS. Early studies showed that patients with IC/BPS had high rates and titers of autoantibodies (antinuclear antibodies and anti‐urothelial antibodies) in their serum and urine [82, 83, 84, 85, 86]. Early studies also showed the overexpression of HLA‐DR molecules, a high‐risk factor for various autoimmune diseases, in the urothelium of patients with IC/BPS [87, 88, 89]. Studies further showed that HIC bladder tissues contained a large number of plasma cell infiltrations and abundant MHC risk gene variants [66, 71]. It has been reported that IC/BPS patients can co‐present systemic autoimmune diseases, such as Sjögren's syndrome (SS), systemic lupus erythematosus (SLE), bronchial asthma, rheumatoid arthritis (RA), and inflammatory bowel disease (IBD) [90, 91]. Conversely, patients with systemic autoimmune diseases, such as SS and SLE, can frequently accompany irritative bladder disorders presenting the IC/BPS symptoms [92, 93]. A study showed that both HIC and BPS can be associated with some type and degree of allergies but RA and IBD occurred more often in HIC than in BPS [90]. Furthermore, female preponderance, a typical epidemiological feature of systemic autoimmune disorders, is a well‐known feature of IC/BPS patients [86]. Collectively, all these findings strongly suggest the possible autoimmune nature of IC/BPS, especially HIC. However, this hypothesis remains controversial as specific autoantigens have not yet been identified in IC/PBS patients.

Recent studies indicated that the skewing of immune responses toward the T helper (Th)1/Th17 axis is responsible for the development and progression of inflammatory and autoimmune diseases, in which IFN‐γ (a major Th1 cytokine) and IL‐17 (a major Th17 cytokine) coordinately play an important role [94]. IFN‐γ can play both a protective and pathogenic role in the immune responses, depending on the specific circumstances. In the context of autoimmune inflammation, IFN‐γ affects the disease severity through its ability to promote T cell differentiation, B cell immunoglobulin (Ig) class switching, and IgG Fc receptor activation [95, 96]. Aberrant IFN‐γ expression has been reported in various inflammatory and autoimmune diseases including SS, SLE, multiple sclerosis (MS), RA, type‐1 diabetes, psoriasis, and dermatomyositis (DM). Previous studies in murine models of SS and SLE supported the potential of IFN‐γ as a therapeutic target for autoimmune diseases, as elevated IFN‐γ levels were observed during peak autoimmune inflammation at the tissue‐specific sites [95]. Treatment with anti‐IFN‐γ monoclonal antibody (mAb), soluble IFN‐γ receptor (IFNγR), or cDNA encoding IFNγR reduced the serum level of IFN‐γ and the autoimmune manifestations in the animal models [95, 97]. In line with these studies, we recently identified the overexpression of IFN‐γ and IL‐17 genes in human HIC bladder tissues [53, 98], providing potential therapeutic targets for the human disease.

## 4 Experimental Autoimmune Cystitis Models

Since autoimmune inflammation is suggested to play a significant role in subgroups of IC/BPS patients, various EAC models have emerged since the early 1990s (Table 3). These EAC models can be generated in rodents through (i) Immunization with syngeneic bladder homogenate; (ii) Immunization with recombinant UPK or UPK peptide; (iii) Transgenic technology that enables the urothelial expression of the well‐defined model antigen OVA as a self‐antigen. Currently, the EAC models are being used in HIC research because these models can closely mimic the key features of human HIC such as chronic bladder inflammation manifested by dense infiltrating mononuclear cells (e.g., T and B lymphocytes and mast cells), bladder tissue damages (e.g., increased urothelial permeability, increased vascularity, mucosal hyperemia, and stromal edema), pelvic/bladder pain, and/or voiding dysfunction [31, 32, 33, 34, 35, 36, 37, 38, 39, 40, 41, 42, 43, 44, 45, 46, 47, 48, 49, 50, 51, 52, 53]. We have long been interested in the development of transgenic EAC models and the application of these animal models to the study of IC/BPS including HIC.

**TABLE 3:** Experimental autoimmune cystitis models.

Species	Strain	Induction method	Autoantigen	References
Mouse	Balb/cAN	Immunize with syngeneic bladder homogenate	Unknown	[31, 32]
Mouse	SWXJ	Immunize with syngeneic bladder homogenate	Unknown	[33, 34]
Rat	Sprague–Dawley	Immunize with syngeneic bladder homogenate	Unknown	[35]
Rat	Lewis	Immunize with syngeneic bladder homogenate	A 12‐kDa protein	[36]
Mouse	SWXJ	Immunize with recombinant UPK II	UPK II	[39]
Mouse	Balb/c‐Fcgr2b^−/‐^Pdcd1^−/−^	Spontaneous	UPK IIIa	[44]
Mouse	Balb/c	Immunize with UPK3A_65–84_ peptide	UPK 3A	[40, 41, 42]
Mouse	C57BL/6	Immunize with syngeneic bladder homogenate	Unknown	[37, 38]
Mouse	C57BL/6	Immunize with TRPM8 T2 peptide	TRPM8	[43]
Mouse	URO‐OVA	Adoptive transfer of OVA‐specific CD8^+^ or CD4^+^ T cells	Transgenic OVA	[45, 46, 47, 48, 49, 50]
Mouse	URO‐OVA	Adoptive transfer of OVA‐sensitized splenocytes	Transgenic OVA	[52, 53]

Abbreviations: OVA, ovalbumin; TRPM8, transient receptor potential melastatin family member 8; UPK, uroplakin; URO, uroplakin II gene promoter.

### 4.1 The URO‐OVA Model

Over the past two decades, we have developed a prototype transgenic EAC model (URO‐OVA) and a panel of its derived EAC models, and used them for mechanistic studies and therapeutic development of IC/BPS [45, 46, 47, 48, 49, 50, 51, 52, 53]. URO‐OVA mice express a membrane form of the model antigen OVA as a self‐antigen on the bladder urothelium driven by the uroplakin II gene promoter [45], and develop autoimmune‐based bladder inflammation upon introduction of OT‐I CD8^+^ T cells that express the transgenic T cell receptor (TCR) specific for H2‐K^b^/OVA_257‐264_ epitope [45, 99]. We have demonstrated that the bladder urothelial lining can present self‐antigen to the immune system and induce bladder autoimmune responses in URO‐OVA mice. This model system provides a useful tool for studying immune cell tolerance, activation, and autoimmune responses in the bladder mucosal immunology. In addition, we have observed histologically significant chronic bladder inflammation in URO‐OVA mice that received OT‐I CD8^+^ T cell transfer, characterized by extensive mononuclear cell infiltration (including lymphocytes and mast cells), increased vascularity, mucosal hyperemia, and stromal edema, with the inflammation peaking at 7–14 days [45, 47, 48]. Furthermore, the inflamed bladder expresses proinflammatory cytokines and chemokines (e.g., IFN‐γ, TNF‐α, IL‐6 and MCP‐1), neurotransmitters (e.g., substance P), neurotrophic factors (e.g., NGF), and angiogenic factors (e.g., VEGF) at the mRNA and/or protein levels [45, 47, 48]. Along with the changes in bladder histopathology and gene expression, cystitis‐induced URO‐OVA mice exhibit increased pelvic/bladder nociceptive responses and irritative voiding symptoms, such as increased urinary frequency and decreased maximum volume voided per micturition [46, 49, 51]. Therefore, the URO‐OVA model reproduces the key clinical features seen in subgroups of IC/BPS, providing a unique model for IC/BPS research.

In addition to CD8^+^ T cells, CD4^+^ T cells can also induce EAC in URO‐OVA mice. We have observed that the adoptive transfer of OT‐II CD4^+^ T cells that express a transgenic TCR specific for the I‐A^b^/OVA_323–339_ epitope induced EAC in URO‐OVA mice [50]. The OT‐II CD4^+^ T cell‐induced EAC was similar to the OT‐I CD8^+^ T cell‐induced EAC, including dense mononuclear cell infiltration, increased vascularity, mucosal hyperemia, and stromal edema in the inflamed bladder. We have further observed that OVA‐specific CD4^+^ T cells could induce EAC in URO‐OVA mice depleted of CD8^+^ T cells. Our observation indicated that CD4^+^ T cells can function as direct effector cells and induce EAC, independent of CD8^+^ T cells, in URO‐OVA mice.

Our early study demonstrated the responsiveness of the URO‐OVA model to intravesical administration of dimethyl sulfoxide (DMSO) [48], one of the mainstays in the current pharmacological treatment of IC/BPS [100, 101]. The DMSO treatment significantly reduced bladder inflammation and gene expression of IFN‐γ, TNF‐α, IL‐6, MCP‐1, and NGF in cystitis‐induced URO‐OVA mice. We observed that DMSO impaired effector CD8^+^ T cell infiltration and viability in vivo and in vitro [48]. In addition, we tested RDP58, a MAP kinase inhibitor [102], for treating chronic bladder inflammation in the URO‐OVA model. We observed that, compared with the control group, intravesical administration of RDP58 in cystitis‐induced URO‐OVA mice significantly reduced bladder inflammation and gene expression of TNF‐α, NGF, and substance P [47].

### 4.2 The URO‐OVA Derived Models

**FIGURE 2:**
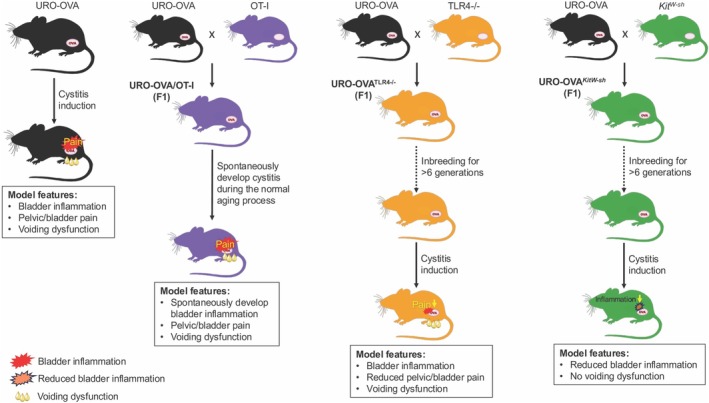
Generation, cystitis development and model features of three URO‐OVA derived models (URO‐OVA/OT‐I, URO‐OVA^TLR4−/−^ and URO‐OVA^*Kit/W‐sh*^). The model features reflect the phenotypic and functional changes in these three URO‐OVA derived models after the development of cystitis. The model features of cystitis‐induced URO‐OVA mice are included for comparison.

We have created three URO‐OVA derived models, URO‐OVA/OT‐I, URO‐OVA^*TLR4−/−*^ and URO‐OVA^*Kit/W‐sh*^ (Figure 2). The URO‐OVA/OT‐I model was generated through crossbreeding between URO‐OVA mice and OT‐I mice that express transgenic CD8^+^ TCR specific for H2‐K^b^/OVA_257‐264_ epitope. The F1 generation acquires both urothelial OVA expression and endogenous OVA‐specific CD8^+^ T cells and thus can spontaneously develop bladder autoimmune inflammation during the normal aging process [45, 48, 51]. URO‐OVA/OT‐I mice developed histological bladder inflammation at 4 weeks of age, peaked at 8 weeks of age, and persisted for up to 20 weeks of age tested. Like the URO‐OVA model, the URO‐OVA/OT‐I model exhibits dense mononuclear cell infiltration, increased vascularity, mucosal hyperemia, and stromal edema in the inflamed bladder, along with pelvic/bladder pain and voiding dysfunction. Also, like the URO‐OVA model, the URO‐OVA/OT‐I model showed a responsiveness to intravesical DMSO treatment [48]. Therefore, this animal model provides a useful tool for studying bladder autoimmune processes and for developing bladder autoimmune interventions.

The URO‐OVA^*TLR4−/−*^ model is a TLR4‐deficient URO‐OVA model that preserves the expression of urothelial OVA but lacks TLR4 expression [49]. The URO‐OVA^*TLR4−/−*^ model was generated through crossbreeding between URO‐OVA mice and TLR4^−/−^ mice, a line genetically deficient in the *TLR4* gene, followed by inbreeding for over 6 generations. We used the URO‐OVA^*TLR4−/−*^ model to investigate the role of TLR4 in chronic cystitis pain, which was implicated in our previous human studies [103, 104, 105]. Interestingly, we observed that, compared to control cystitis‐induced URO‐OVA mice, cystitis‐induced URO‐OVA^*TLR4−/−*^ mice developed similar bladder inflammation and voiding dysfunction but with significantly reduced pelvic/bladder nociceptive responses [49]. We next treated cystitis‐induced URO‐OVA mice with intravenous TAK‐242, a TLR4 selective inhibitor [106], and observed significantly reduced pelvic/bladder pain in the treated mice [49]. Our study demonstrated the important role of TLR4 in chronic cystitis pain seen in patients with IC/BPS.

The URO‐OVA^*Kit/W‐sh*^ model is a mast cell‐deficient URO‐OVA model that preserves the expression of urothelial OVA but lacks mast cells [46]. The URO‐OVA^*Kit/W‐sh*^ model was generated through crossbreeding between URO‐OVA mice and *Kit* ^*W‐sh*^ mice, a line genetically deficient in the *Kit* ^*W‐sh*^ gene (a key regulator of the mast cell lineage) [107], followed by inbreeding for over 6 generations. We observed that compared with control cystitis‐induced URO‐OVA mice, cystitis‐induced URO‐OVA^*Kit/W‐sh*^ mice developed reduced bladder inflammation with no voiding dysfunction [46]. Our study provided evidence for the role of mast cells in the development of bladder autoimmune inflammation and related voiding dysfunction.

### 4.3 The HIC‐Like URO‐OVA Model

Besides T lymphocytes, B lymphocytes are implicated to play a pivotal role in the pathophysiology of HIC [10, 66, 73]. To further improve the URO‐OVA model and make it more closely resemble the simulation of HIC, we recently modified the cystitis induction method and developed a more HIC‐like URO‐OVA model. To generate both antigen‐specific T and B lymphocytes for cystitis induction, we immunized C57BL/6 mice with OVA protein emulsified with complete Freund's adjuvant (CFA). After 14 days, OVA‐sensitized splenocytes (a mixture of T and B cells) were collected from OVA immunized mice and transferred into URO‐OVA mice for cystitis induction. This modified URO‐OVA model developed chronic bladder inflammation, pelvic pain and voiding dysfunction at Days 7–28 with a peak at Day 21 after cystitis induction [52]. Like the previous URO‐OVA model that used OT‐I CD8^+^ or OT‐II CD4^+^ T cells for cystitis induction, this new URO‐OVA model developed remarkable bladder histopathology manifested by profound mononuclear cell infiltration, increased vascularity, mucosal hyperemia, and stromal edema [52]. Notably, this new URO‐OVA model showed both dense CD3‐positive T cells and CD19‐positive B cells within the lymphoid follicle‐like structures in the inflamed bladder, similar to findings in human HIC [52, 62]. Also, like the previous URO‐OVA model, this new URO‐OVA model expressed elevated levels of mRNAs for multiple proinflammatory and nociceptive factors, such as IFN‐γ, TNF‐α and substance P, in the inflamed bladder [52].

Since human HIC bladder can express high levels of IFN‐γ protein and mRNA [53], we investigated whether IFN‐γ could serve as a potential therapeutic target for HIC. We used a synthetic anti‐mouse IFN‐γ DNA aptamer to treat this HIC‐like URO‐OVA model. URO‐OVA mice were induced for cystitis by adoptive transfer of OVA‐sensitized splenocytes at Day 0. Subsequently, starting from Day 0 after cystitis induction, anti‐mouse IFN‐γ DNA aptamers were injected intravesically every other day for a total of 12 times. During treatment, mice were analyzed for pelvic pain and voiding dysfunction. At the end of the experiment, bladder histology and gene expression were analyzed. We observed that, compared with the control group, URO‐OVA mice treated with anti‐mouse IFN‐γ DNA aptamers showed significantly reduced bladder inflammation, increased pelvic sensory threshold, and decreased urinary frequency [53]. Furthermore, compared with the control group, these URO‐OVA mice treated with anti‐mouse IFN‐γ DNA aptamers showed reduced mRNA levels of IFN‐γ, TNF‐α, substance P, and NGF [53]. Our study provides a novel HIC‐like mouse model and supports the role of IFN‐γ in the development and progression of HIC.

## 5 Conclusion

Currently, there are a wide variety of animal models available for IC/BPS research, and each animal model can reproduce certain key features of the human disease. Due to the diverse clinical phenotypes of IC/BPS and the high heterogeneity of patient populations, selecting a suitable animal model that can closely mimic a specific condition of IC/BPS for research is challenging. At present, IC/BPS is categorized into two subtypes, that is, HIC and BPS. These two IC/BPS subtypes have different pathological mechanisms, but their clinical symptoms overlap. Today, animal models of EAC are used in HIC research, while animal models associated with increased urothelial permeability, psychological/physical stress, and pelvic cross‐organ sensitization are used in BPS research. The URO‐OVA model is a unique transgenic EAC model. It is stable and reproducible due to its genetic stability. It develops chronic bladder inflammation, pelvic/bladder pain, and voiding dysfunction seen in human HIC patients. Furthermore, the URO‐OVA model responds to treatment with DMSO and specific inhibitors such as TLR4, MAP kinase and IFN‐γ inhibitors. Hence, the URO‐OVA model provides a useful tool for mechanistic studies and therapeutic development of HIC. Although EAC models can mimic the pathophysiology of HIC, the true pathogenic autoantigen in human HIC has not yet been identified. Currently, no animal models can precisely and thoroughly mimic human IC/BPS including HIC. Therefore, continued efforts to develop subtype‐specific animal models are crucial for our improved understanding of this undefined and mysterious human disease.

## Author Contributions

**Yoshiyuki Akiyama:** conceptualization, data curation, writing – review and editing, funding acquisition. **Yi Luo:** writing – original draft, conceptualization, data curation, funding acquisition.

## Funding

This work was supported by the Office of the Assistant Secretary of Defense for Health Affairs through the Chronic Pain Management Research Program (CPMRP) [Grant number HT94252310979] (to YL) and the KAKENHI Grants‐in‐Aid from the Japanese Society for the Promotion of Science (JSPS) [Grant number 25K02771] (to YA).

## Ethics Statement

The authors have nothing to report.

## Consent

The authors have nothing to report.

## Conflicts of Interest

The authors have no relevant financial interests to disclose regarding the materials discussed in the manuscript. Yoshiyuki Akiyama is an Editorial Board member of the International Journal of Urology and a co‐author of this article. To minimize bias, he/she was excluded from all editorial decision‐making related to the acceptance of this article for publication.

## Data Availability

The authors have nothing to report.
